# The emergence of sexually transmissible Shigella flexneri serotype 1b between 2019 and 2024 in England: a descriptive epidemiological study

**DOI:** 10.1099/mgen.0.001495

**Published:** 2025-09-15

**Authors:** Ailie Robinson, Ella V. Rodwell, Neville Q. Verlander, Hannah Charles, Kate S. Baker, Marie Anne Chattaway, Claire Jenkins

**Affiliations:** 1 Gastrointestinal Bacteria Reference Unit, UK Health Security Agency, London, UK; 2 Gastrointestinal Infections & Food Safety (One Health), UK Health Security Agency, London, UK; 3 Statistics, Modelling and Economics Department, UK Health Security Agency, London, UK; 4 Blood Safety, Hepatitis, Sexually Transmitted Infections (STIs) and HIV Division, UK Health Security Agency, London, UK; 5 Department of Genetics, University of Cambridge, Cambridge, CB2 3EH, UK; 6 NIHR HPRU in Gastrointestinal Infections, Universities of East Anglia & Newcastle, Norwich, UK

**Keywords:** molecular typing, sexual transmission, *Shigella flexneri*, surveillance, travellers’ diarrhoea

## Abstract

**Background.** *Shigella* species are pathogenic bacteria that cause gastrointestinal symptoms ranging from mild watery diarrhoea to bacillary dysentery. Transmission is faecal–oral and historically associated with international travel. Recently, sexual transmission has been documented among gay, bisexual and other men who have sex with men (GBMSM). Through routine surveillance, we observed an increase in notifications of *S. flexneri* serotype 1b. We investigated the emergence of this serotype and examined possible drivers of transmission.

**Methods.** We used historical data and whole-genome sequencing data from *S. flexneri* 1b isolates submitted to the United Kingdom Health Security Agency (UKHSA) to determine the relatedness of isolates and describe the population structure using phylogenetics. We tested for associations with possible epidemiological, biological and genetic drivers.

**Results.** Between 1 January 2004 and 30 June 2024, 1,672 isolates of *S. flexneri* 1b were identified. Prior to 2019, there was a median of 12.5 [interquartile range (IQR) 10–17] notifications per quarter, rising to a median of 39.5 (IQR 23–58) notifications from 2019 to 2024. The rise was predominantly among adult males, consistent with patterns seen in prior sexually transmitted shigellosis epidemics among GBMSM. Unlike previous outbreaks of shigellosis among GBMSM, the emergence of *S. flexneri* 1b showed no evidence of an association with the acquisition of antimicrobial resistance determinants.

**Conclusions.** Shigellosis can have severe clinical outcomes, and the repeated emergence of *Shigella* variants among GBMSM highlights the significance of the sexual transmission pathway. Continued surveillance of *Shigella* subtypes is necessary to inform public health interventions aimed at preventing sexual transmission of enteric pathogens in the GBMSM community.

**Impact Statement**

*Shigella flexneri* is a causative agent of shigellosis.In the UK, *S. flexneri* was historically associated with travel to endemic regions and community outbreaks in schools and pre-school settings. Since 2010, different serotypes of *S. flexneri* have caused sequential epidemics of shigellosis among gay, bisexual and other men who have sex with men (GBMSM) driven by acquisition of antimicrobial resistance to macrolides, fluoroquinolones and, more recently, the third-generation cephalosporins. *S. flexneri* serotype 1b emerged as a cause of sexually transmitted shigellosis during the severe acute respiratory syndrome coronavirus 2 pandemic and has persisted in the GBMSM community ever since. Despite this persistent phenotype, the GBMSM *S. flexneri* 1b clade is not associated with the same characteristics driving the transmission of isolates belonging to previously described GBMSM clades, specifically plasmid-encoded resistance to azithromycin and the third-generation cephalosporins, acquisition of mutations encoding resistance to fluoroquinolones and phage-plasmid encoded *metG*. Intervention strategies should consider that the emergence and persistence of *Shigella* species circulating concurrently in the GBMSM community may be influenced by a variety of different factors, including, but not exclusively, resistance to current antimicrobial treatment regimens.

## Data Summary

Fastq files have been submitted to the National Center for Biotechnology Information. All data can be found under BioProject no. PRJNA315192 and in Table S1.

## Introduction

The shigellae are facultatively intracellular bacteria that invade the colonic mucosa of humans [1]. This causes shigellosis, an infection characterized by symptoms ranging from mild watery diarrhoea to classic bacillary dysentery, bloody or mucous-containing diarrhoea, fever, nausea and abdominal cramps [2]. In severe cases, shigellosis can be life-threatening and may require treatment with antimicrobials [3]. Shigellae primarily infect humans, and there is no known animal reservoir. Transmission is faecal–oral, and the infectious dose of *Shigella* species is low (10–100 bacteria), so *Shigella* is easily transmitted between people by direct contact or via faecally contaminated food or water [4]. Found globally, shigellae commonly cause childhood diarrhoeal disease in resource-limited settings where the associated disease burden is high [5]. Of the four species of *Shigella*, historically in the UK, isolation of *Shigella dysenteriae*, *Shigella flexneri* or S*higella boydii* has been classically associated with overseas travel to endemic regions (e.g. Africa, Asia and Latin America), while *Shigella sonnei* was transmitted endemically, especially in school or nursery school settings [4]. In addition to this worldwide epidemiology as a diarrhoeal pathogen, robust evidence has emerged of sexual transmission of *S. sonnei* and *S. flexneri* among gay, bisexual and other men who have sex with men (GBMSM) [6,8].

Sexual transmission of *Shigella* species occurs via direct oral–anal contact, via oral–genital contact when genitalia are contaminated with faecal bacteria or indirect contact via fingers or fomites [9]. Transmission is promoted within GBMSM sexual networks of individuals with similar risk profiles, which can include multiple sexual partners, chemsex and attendance at sex-on-premises venues [10,11]. Between 37–75% of GBMSM shigellosis cases are living with human immunodeficiency virus (HIV) [6,11,13]. Although first reported in San Francisco in 1974 [14], the first GBMSM-associated (GBMSM-A) shigellosis outbreak in the UK was described in 2004 [15]. Over the last decade, notifications of GBMSM-A *S. flexneri* and *S. sonnei* in the UK have increased [4].

Globally, the number of outbreaks of shigellosis in GBMSM has increased over the last two decades [9] and these epidemics are a significant public health concern. Sexually transmissible shigellosis is often associated with multidrug-resistant (MDR) and extensively drug-resistant (XDR) strains of *S. flexneri* and *S. sonnei*, exhibiting resistance to azithromycin, ciprofloxacin and the third-generation cephalosporins [16,17]. The World Health Organization classifies ciprofloxacin and third-generation cephalosporin-resistant *Shigella as* a priority AMR pathogen [18].

The need to understand and monitor the emergence of MDR and XDR *Shigella* species and serotypes, along with their associated traits, is clearly a priority for clinical management and public health in the UK. Since the severe acute respiratory syndrome coronavirus 2 (SARS-CoV-2) pandemic, through routine surveillance, we have observed steady increases in notifications of *S. flexneri* serotype 1b. In this genomic epidemiological study, we aimed to investigate the recent emergence of *S. flexneri* 1b and ascertain risk factors and routes of transmission. We further aimed to investigate the genomic epidemiology of 1b isolates, examining for clades that may be associated with emergence, e.g. in sexual transmission networks. Finally, we tested for relationships between emerging subtypes and epidemiological and biological factors that may drive emergence, including the sex ratio of patients, their travel history, geographical location and antimicrobial resistance genes carried by the *S. flexneri* 1b isolates.

## Methods

### Study design and data collection

We identified all *S. flexneri* serotype 1b isolates in the Gastrointestinal Bacteria Reference Unit (GBRU) database, maintained by the UK Health Security Agency (UKHSA), between 1 January 2004 and 30 June 2024. Pathogen surveillance in the GBRU occurs through the monitoring of clusters of genetically related isolates sent from hospitals and other diagnostic laboratories for confirmation and typing. In brief, isolates are sequenced using whole-genome sequencing (WGS), assigned a SNP address that describes their position in the population structure, and clusters are defined by differences in SNP addresses.

Referral forms are used to submit presumptive *Shigella* isolates to GBRU. These include basic questions regarding the specimen/patient (patient information, travel history and initial serotyping). Any data on referral forms are entered into the UKHSA laboratory information system and linked to all laboratory findings (including WGS) in the Gastrointestinal Data Warehouse (GDW). The GDW was the source of data for this study. Sex is requested on the referral form. Patient age was categorized as follows: <16 years, 16–34 years, 35–64 years, >65 years; these groupings were chosen to represent children and young, middle-aged and older adults. Because of the lack of sexual orientation information available in this dataset, we used a proxy indicator of cases that might be attributed to sexual transmission among GBMSM, defined as cases among male adults (>16 years) without a history of travel or where travel history was unknown [presumptive men who have sex with men (MSM)]. Travel history is requested on the referral form. In many instances, travel history is left blank as a proxy for writing ‘no’, and as such, ‘no’ cannot be differentiated from unknown. Travel location when stated was allocated to high risk for *Shigella* transmission (Africa, the Middle East, Latin American countries including the Caribbean and Guyana) or low risk (Europe), as well as neither (country of neither high nor low risk category) or missing. Patient location (postcode) and UKHSA regions are routinely recorded in the patient information. We used quarterly time periods for finer temporal resolution; these are quarter 1 (Q1, 1) January–March, quarter 2 (Q2, 2) April–June, quarter 3 (Q3, 3) July–September and quarter 4 (Q4, 4) October–December.

### Illumina whole-genome sequencing, genomic analysis and gene detection

Microbiological typing, including confirmation of the species and the serotype, was performed at UKHSA using WGS (Table S1, available in the online Supplementary Material) [19,20]. DNA was extracted for sequencing on an Illumina HiSeq 2500 instrument. Identification to the species level was based on kmer identification [21]. Genome-derived serotyping and AMR determinant profiling were performed using the GeneFinder tool (https://github.com/phe-bioinformatics/gene_finder), run using default parameters. For serotyping, a reference database containing the gene sequences encoding the 12 O-antigen synthesis or modification genes was constructed and then used to determine serotype as previously described [20].

For AMR determinant profiling, genes were defined as present if they represented 100% of the reference sequence with greater than 90% nucleotide identity [22]. The reference database for AMR determinants can also be found in the GeneFinder GitHub repository (https://github.com/phe-bioinformatics/gene_finder/tree/master/refs). The prevalence of resistance to streptomycin, tetracycline, sulphonamides and trimethoprim in *Shigella* species is high, and the trends have been consistent for many years [17,23, 24]. We, therefore, focused our AMR analysis on the presence of genomic markers of resistance to azithromycin (*ermB* and/or *mphA*), ciprofloxacin (mutations in *gyrA* and/or *parC*) and the third-generation cephalosporins (*bla*_CTX-M_ variants), as the trends in resistance to these clinically relevant classes of antimicrobials fluctuated during the study period.

We conducted SNP typing on the *S. flexneri* isolates. We applied single-linkage hierarchical clustering at seven descending thresholds of SNP distances (Δ250, Δ100, Δ50, Δ25, Δ10, Δ5 and Δ0) as previously described. That clustering resulted in a discrete 7-digit code, where each number represents the cluster membership at each descending SNP distance threshold. For *Shigella* spp. surveillance, we designated isolates that cluster at the 10 SNP threshold t10.X.

### Phylogenetic tree construction

A phylogenetic analysis was conducted of all *S. flexneri* serotype 1b genomes (*n*=1,036) for whom SNP address data were available (i.e. from September 2015). SnapperDB v0.2.8 holds variant positions of genomes of *S. flexneri* to reference 2457T (AE014073.1). A whole-genome alignment of genomes representing the 100 SNP level was generated and Gubbins v2.00 was used to mask recombinant regions. A soft-core genome alignment of *S. flexneri* serotype 1b genomes was then generated from SnapperDB v0.2.8 with recombinant regions masked. A maximum likelihood phylogeny was constructed using IQ-TREE v2.0.4. The phylogenetic tree was then annotated (iTOL v6).

### Statistical analysis methods

Statistical analysis was performed on isolates received since 2015 for whom WGS data were available, and isolates were phylogenetically mapped to either travel-associated (Travel-A) lineages or GBMSM-A clades. Further, from this point, antimicrobial resistance genes (ARG) data were available. Season was defined for analysis as spring (March/April/May), summer (June/July/August), autumn (September/October/November) and winter (December/January/February). The SARS-CoV-2 epidemic timescale was modelled as pre-, during (16/03/2020-08/12/2020, the latter date being initiation of vaccine roll-out) and post-Coronavirus disease (COVID) pandemic (thereafter to 09/07/2024). Data were cleaned and checked for outliers and implausible values; these were corrected if possible or set to missing (two observations with missing sex and three observations with missing date of birth were dropped). There were many missing observations in the travel exposure (*n*=679/1,027). For the mixed effects logistic regression only, we assumed that missing travel information was indicative of no travel. Of those who had travelled (*n*=181), some did not provide a location (*n*=15) and could not be assigned to a risk group. No other variables had missing data, and as such, a complete-case analysis was performed. There were repeated measures on some individuals (*n*=57), which were left in the dataset and accounted for in the analysis by using individual as random effect.

A multivariable mixed effects logistic regression was used to investigate the association of measured variables with the GBMSM-A clades. Individual was the random effect, exposure variables were fixed effects and a linear spline was used with a knot at the year 2020 (year was the time unit) to account for trend. The variance component was removed as it was so small, and purely fixed effect models subsequently fitted. Variables which were inestimable or unstable were removed one at a time, before testing the remaining variables for interactions one at a time with COVID time periods (pre-, during or post-COVID) to determine whether the effect varied across these periods. Interactions were removed if not significant (likelihood ratio test and 5% level) or poorly estimated before adding another.

The Fisher’s exact test was used to test whether multiple isolates from the same patient were more prevalent in either Travel-A lineages or GBMSM-A clades. All analysis was performed in STATA 18.0.

### Ethics

The UKHSA has the authority to handle patient data for public health monitoring and infection control under section 251 of the UK National Health Service Act of 2006 (previously section 60 of the Health and Social Care Act of 2001) [25]. For this reason, no individual patient consent was sought or required.

## Results

### The emergence of serotype 1b

**Fig. 1.**
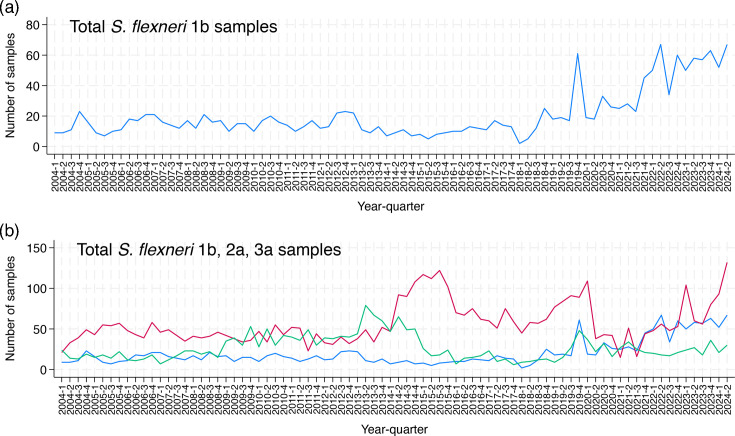
Temporal distribution of cases of *S. flexneri* serotype 1b (**a**) and 1b, 2a and 3a (**b**) per quarter. Colours in figure (b) are blue, 1 b; red, 2a; and green, 3a*.*

Between 1 January 2004 and 30 June 2024, 1,672 isolates of *S. flexneri* 1b were identified by GBRU. From 2004 to 2018, the prevalence of 1b remained stable, with a median of 12.5 [interquartile range (IQR) 10–17] isolates per quarter. Between 2019 and 2024, there was a steady rise in notifications of 1b, with a median of 39.5 (IQR 23–58) isolates per quarter (Fig. 1a). Historically, epidemics of *S. flexneri* serotypes 3a and 2a occurred in sequential waves with the *S. flexneri* 3a epidemic emerging in 2009 and the *S. flexneri* 2a epidemic in 2014 (Fig. 1b). In 2020 and 2021, for the first time, all three serotypes (1b, 2a and 3a) were approximately equally prevalent, after which cases of 1b and 2 a coincidentally increased (Fig. 1b).

### Epidemiological context of 1b

**Fig. 2.**
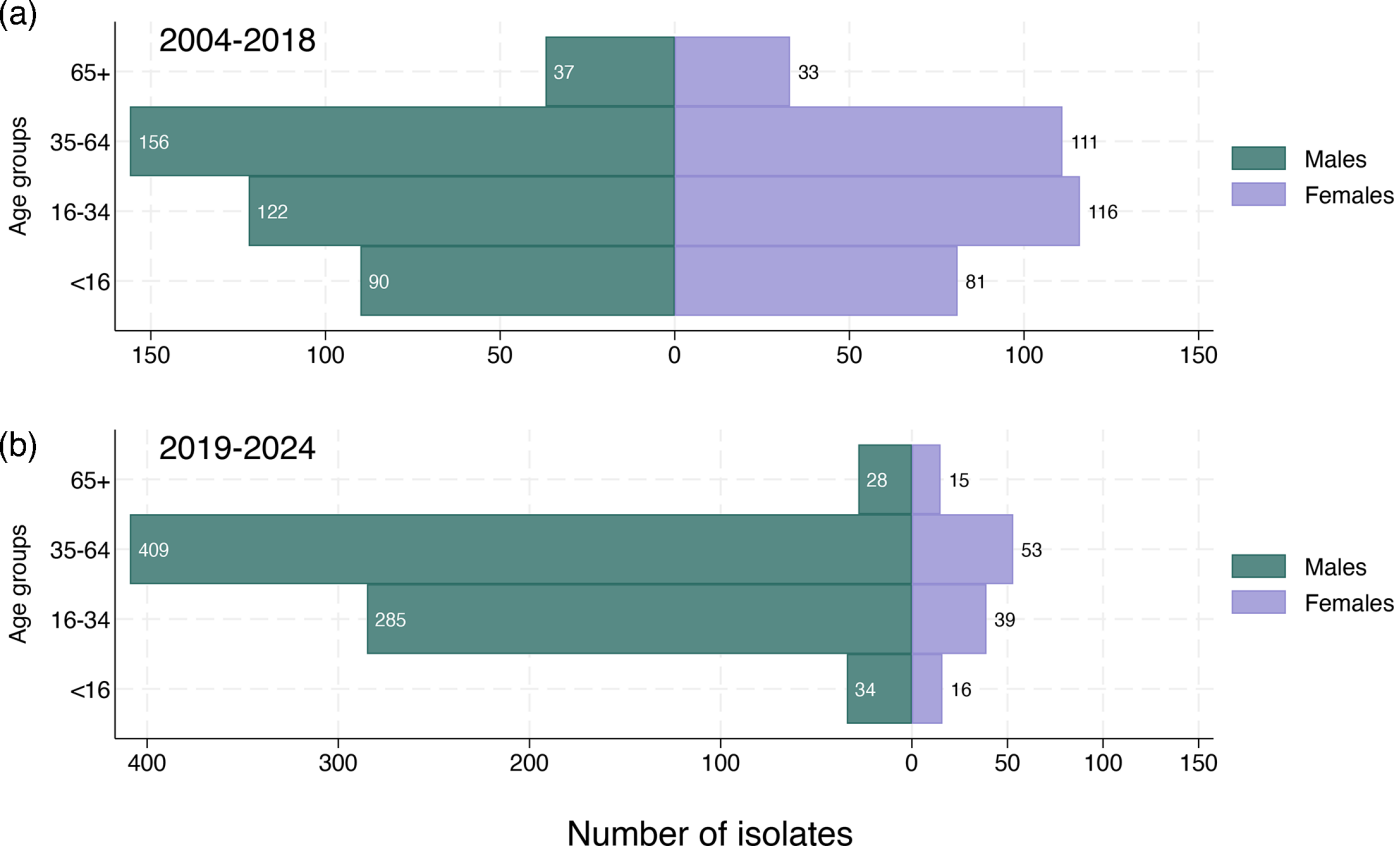
Population pyramids showing the age and sex distribution of the study population from 2004 to 2018 and from 2019 to 2024. Isolate number shown in bar end. Note *x*-axis scale difference between panels (a) and (b).

From 2019, we observed a dramatic rise in the proportion of patients submitting *S. flexneri* 1b isolates that were male (Fig. 2). Before 2019, patients were 45.7% female and 54.0% male (362 female/428 male/3 unknown); from 2019 to 2024, patients were 14.0% female and 86.0% male (121 female/756 male/2 unknown). These changes are illustrated by the changing male/female sex ratio over the study period, from a pre-2019 average of 1: 1 to between 6:1 and 10:1 in the years 2020–2024 (Table 1). The age distribution over the study period remained relatively constant with most cases in the 35–64 age group [before 2019, 265 cases (33.4%); 2019 onwards, 462 cases (53.0%)] (Fig. 2).

**Table 1.** Number of isolates according to demographic category per year. Cells show the number (% in that category)

From 2019, we noted a rise in the number of isolates from patients in London and the North West (before 2019: London, 31.9 % (*n*=250), NW 12.4% (*n*=97); from 2019: London, 42.8% (*n*=375), NW 19.5% (*n*=171) (Table 1).

Travel history information was not provided by 58.7% (953) of patients. However, the number of patients for whom travel history was recorded as ‘yes’ decreased to nearly zero (2%) in 2020, in line with travel restrictions associated with SARS-CoV-2 pandemic control measures (Table 1). The number of notifications of *S. flexneri* 1b continued to rise during this period (Fig. 1a). Among those detailing travel, prior to 2019, the majority (37.1%; *n*=294) specified travel to a high-risk location and only 2 % (*n*=16) specified low-risk (Table 1). From 2019, 7.6% (*n*=67) of individuals stated a travel destination visited high-risk areas, and 2.7% (*n*=24) specified low-risk, with the substantial drop in high-risk travel occurring in 2020 (Table 1).

### *S. flexneri* 1b phylogeny highlights a distinct GBMSM-A clade

**Fig. 3.**
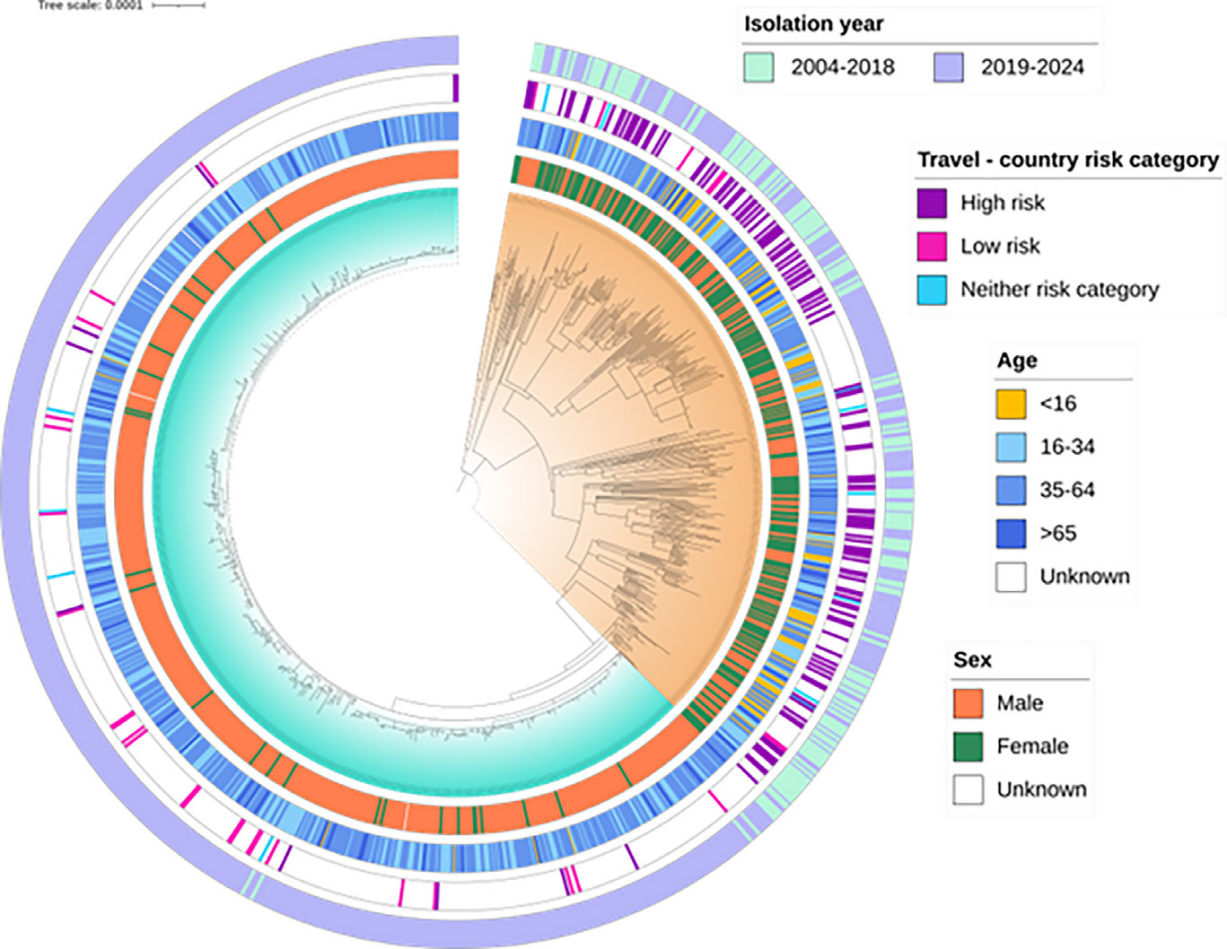
A midpoint rooted maximum likelihood phylogenetic tree showing relatedness between isolates of *S. flexneri* serotype 1b isolated in England between 2004 and 2024. The GBMSM-A clade is highlighted in turquoise, and the Travel-A lineage is highlighted in orange. Epidemiological data describing patient sex, age and travel history, as well as isolate date (year), have been annotated according to inlaid keys. Branches represent substitutions across a 22,985 bp alignment. The reference genome used was 4,522,047 bp in length.

Phylogenetic analysis revealed a genetically conserved clade comprising *S. flexneri* 1b that fell within the same 25 SNP single linkage cluster, designated t25.326, with the majority isolated between 2019 and 2024. This monophyletic group was denoted GBMSM-A. Outside of the t25.326 monophyletic group, the phylogeny was more diverse with longer branches, indicative of greater genetic distances between strains (Fig. 3). These isolates were classified as part of a group denoted as Travel-A lineages, as 119/374 (31.8 %) patients infected with isolates belonging to this group reported travel outside the UK (Fig. 3).

We investigated associations between epidemiological and microbiological factors and the GBMSM-A using mixed effects logistic regression. A total of 1,008 observations, comprising 948 individuals, were available for analysis. The GBMSM-A clade was associated with a higher proportion of male cases [odds ratio (OR) 22.3, 95% confidence interval (CI) 9.62–51.6; *P*<0.001] and a lower proportion of children of less than 16 years (OR 0.06, 95 % CI 0.01–0.31; *P*=0.004) (Table 2).

**Table 2.** Independent associations with GBMSM-A clade, adjusting for trend

Variable	Modifier category	Category	Travel (*N*=365)	MSM (*N*=662)	OR*	95% CI	*P* value†
Sex		Male	195	636	22.3	9.62, 51.6	<0.001
		Female	170	26	1	Baseline	
Age (years)		>65	27	31	0.89	0.21, 3.75	0.004
		35–64	158	362	0.6	0.30, 1.20	
		16–34	99	263	1	Baseline	
		<16	81	6	0.06	0.01, 0.31	
Travel risk group		High risk	116	10	0.05	0.02, 0.16	<0.001
		Low risk	6	23	8.79	0.92, 89.2	
		Neither	7	4	0.19	0.02, 1.68	
		No travel	223	623	1	Baseline	
PHE centre		Anglia	0	0	n.e.	n.e.	0.03
		East Midlands	13	21	1	0.06, 16.5	
		East of England	21	28	0.29	0.03, 3.15	
		London	147	284	0.66	0.08, 5.71	
		North East	6	7	1	Baseline	
		North West	41	151	2.28	0.23, 22.6	
		South East	72	79	0.37	0.04, 3.56	
		South West	18	26	0.47	0.04, 6.17	
		West Midlands	24	19	0.16	0.01, 1.92	
		Yorkshire and Humber	23	43	1.25	0.09, 18.26	
OXA1		Not present	202	28	1	Baseline	0.06
		Present	163	634	4.99	1.04, 24.0	
	Pre-COVID	Not present	225	44	1	Baseline	
		Present	6	2	138	3.39, ∞	
CTX	During COVID	Not present	14	113	3.32	0.76, 14.5	0.008*
		Present	5	1	1.15	0, ∞	
	Post-COVID	Not present	103	490	6.52	1.16, 36.3	
		Present	12	12	1.34	0.06, 32.7	
TEM1		Not present	244	562	1	Baseline	0.06
		Present	121	100	0.24	0.06, 1.06	
qnrS		Not present	261	655	1	Baseline	0.03
		Present	104	7	0.1	0.01, 0.93	
MPHA		Not present	334	547	1	Baseline	0.3
		Present	31	115	2.54	0.49, 13.1	
Tet		Not present	79	18	1	Baseline	0.02
		Present	286	644	0.23	0.07, 0.83	
Sul1		Not present	348	647	1	Baseline	0.96
		Present	17	15	0.8	0.08, 7.48	
Sul2		Not present	216	375	1	Baseline	0.99
		Present	149	287	1.06	0.51, 2.20	
CatA1		Not present	206	17	1	Baseline	<0.001
		Present	159	645	35.6	7.5, 169	
Season		Spring	83	193	1	Baseline	0.16
		Summer	60	157	2.26	0.86, 5.90	
		Autumn	126	175	2.4	0.95, 6.09	
		Winter	96	137	1.09	0.44, 2.70	

*OR compare the odds of being in GBMSM-A clade among individuals in the different variable categories.

†*P* value tests the hypothesis that the OR is equal in different categories of the variable; the smaller the *P* value, the stronger the evidence that the ORs differ between groups.

Although overall, recent increases in the isolation of 1b appear to focus in London and the north west of England (Table 1), there was no GBMSM-A-specific increase in London (OR 0.66, 95% CI 0.08–5.7). However, patients resident in the North West of England (which includes the cities of Manchester and Liverpool) had higher odds of being in the MSM clade than those from the West Midlands (NW OR 2.28, 95% CI 0.23–22.6; WM OR 0.16, 95% CI 0.01–1.92). There was evidence that overall, patient geographical location affected the association with GBMSM-A clade (*P*=0.03) (Table 2).

Those patients who reported travel to destinations that were high risk for *Shigella* species had lower odds to be in the t25.326 clade (OR 0.05, 95% CI 0.02–0.16; *P*<0.001), although there was evidence that those who travelled to low-risk destinations had higher odds to be in the t25.326 clade (OR 8.79, 95% CI 0.92–89.2; *P*<0.001).

### Antimicrobial resistance genes in GBMSM-A clades vs. Travel-A lineages

**Fig. 4.**
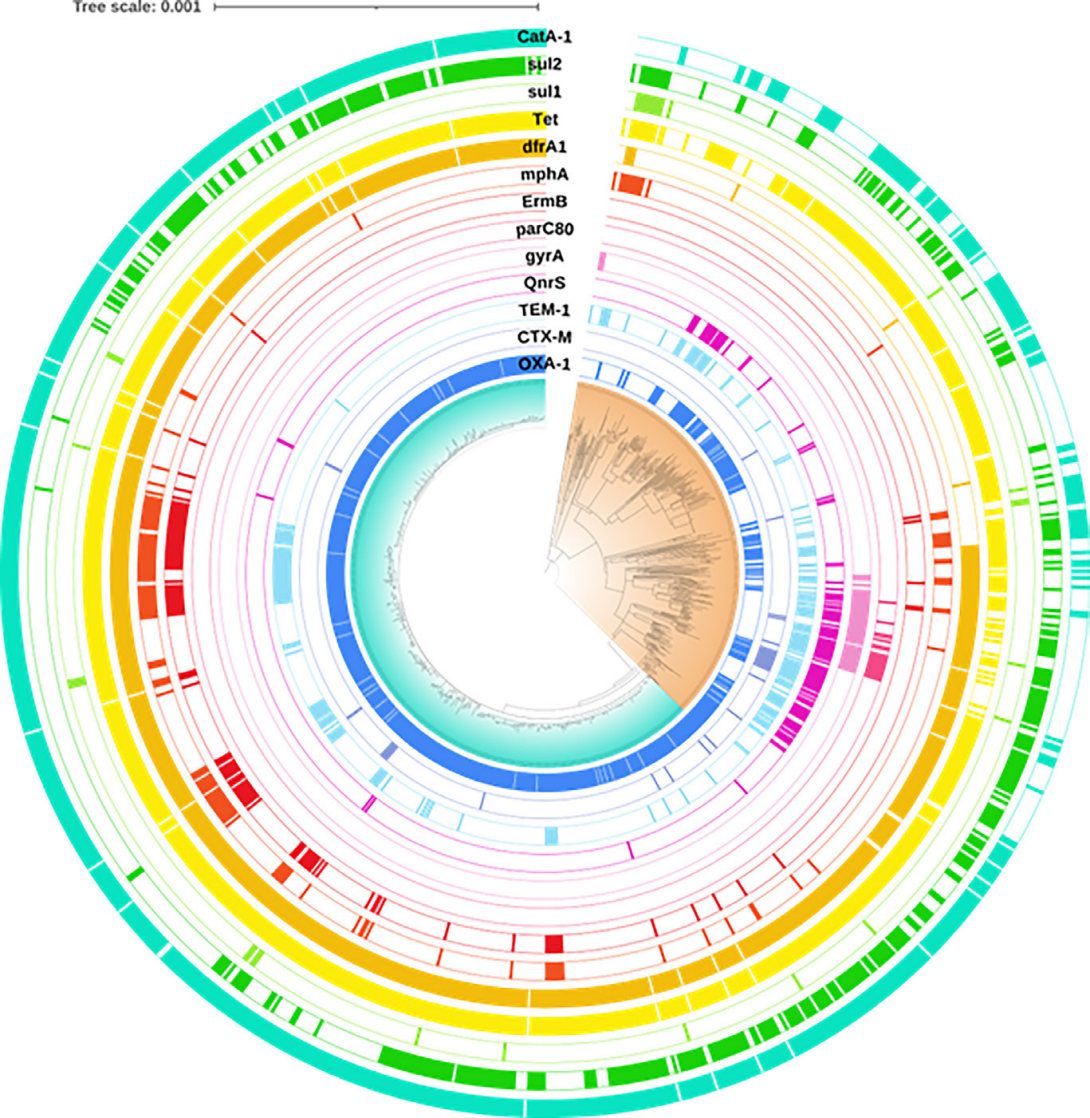
A midpoint rooted maximum likelihood phylogenetic tree showing the GBMSM-A clade (turquoise) and the Travel-A clade (orange) annotated with antimicrobial resistance genes. Branches represent substitutions across a 22,985 bp alignment. The reference genome used was 4,522,047 bp in length.

To investigate possible drivers of the emergence of the GBMSM-A clade, we examined the prevalence of ARG (Fig. 4). Relative to the travel lineages, we observed high prevalence of *bla_OXA-1_* [overall prevalence from 2015: GBMSM-A, 95.8% (*n*=638); Travel-A, 44.8% (*n*=164)] and resistance to chloramphenicol via *catA1* [GBMSM-A, 97.5% (*n*=649); Travel-A, 43.7% (*n*=160)]. Resistance to trimethoprim via *dfrA1* was relatively higher in the GBMSM-A clades [GBMSM-A, 97.5% (*n*=649); Travel-A, 38.5% (*n*=141)], and there were high levels of resistance to tetracycline (via *tet*) in both lineages [GBMSM-A, 97.3% (*n*=648); Travel-A, 78.4% (*n*=287)]. Some resistance to the macrolides, conferred by *ermB* and *mphA*, was present in the GBMSM-A clades but was almost absent in the travel-associated lineages [*ermB*: GBMSM-A, 16.3% (*n*=109); Travel-A, 1.1% (*n*=4) and *mphA*: GBMSM-A, 17.3% (*n*=115); Travel-A, 8.5% (*n*=31)]. Resistance to the fluoroquinolones, including ciprofloxacin (via *qnrS*, or mutations in *gyrA*, or *parC80*), was notably barely present overall and almost absent in the GBMSM-A lineage (Fig. 4). While the extended-spectrum beta-lactamase (ESBL) *bla_CTX_* gene was not frequently detected [GBMSM-A, 2.3% (*n*=15/651); Travel-A, 6.3% (*n*=23/342)], different variants of *bla_CTX-M_* appeared to be clade-associated. Most instances of *bla_CTX-M-27_* were in the GBMSM-A clade [87.5% (*n*=7/8)], while most instances of *bla_CTX-M-15_* were in the Travel-A lineages [80.8% (*n*=21/26)].

Of the ARG, isolates harbouring *bla_OXA-1_* or *catA1* had higher odds of being in the GBMSM-A clade (OR 4.99, 95% CI 1.04–24.0; *P*=0.06; OR 35.6, 95% CI 7.5–169.0; *P*<0.001 respectively) (Table 3). Isolates with *qnrS* were less likely to be in the GBMSM-A clade (OR 0.1, 95% CI 0.01–0.93; *P*=0.03); similarly, there was some evidence that isolates with *bla_TEM-1_* were reduced in GBMSM-A (OR 0.24, 95% CI 0.06–1.06; *P*=0.06). Contrary to our initial observations from the phylogenetic tree, isolates carrying *tet* were less likely to be in the GBMSM-A clade (OR 0.23, 95% CI 0.07–0.83; *P*=0.02) (Table 3). It was not possible to investigate associations for the ARG *gyrA*, *parC80*, *ermB* and *dfrA1*, as small sample sizes prevented reliable estimations from being made. In the pre-COVID time period, *bla_CTX_* was associated with higher odds of being in the GBMSM-A clade; there was no significant association during COVID, and post-COVID, the absence of *bla_CTX_* was associated with higher odds of being in the GBMSM-A clade. This interaction is probably at least partly accounted for by the distribution of the *bla_CTX_* variants. While *bla_CTX-15_* was detected across all three COVID periods [pre-COVID, 26.9% (*n*=7); COVID, 23.1% (*n*=6); post-COVID, 50% (*n*=13)], all eight *bla_CTX-27_* isolates were detected post-COVID (100%).

**Table 3.** Prevalence of individual antimicrobial resistance genes in *S. flexneri* serotype 1b isolates between 2004 and 2024 in the GBMSM-A and Travel-A clades. Cells show percentages (frequency, *n*).

Year												
Antibiotic class	Resistance gene	Clade	2015	2016	2017	2018	2019	2020	2021	2022	2023	2024
	Number of samples of	GBMSM-A	0	2	3	3	33	76	105	168	185	91
	serotype 1b	Travel-A	13	42	52	39	77	19	16	38	43	28
Beta-lactams	*bla*_*OXA-1*_	GBMSM-A	0 (0)	100(2)	100 (3)	100 (3)	100 (33)	97 (74)	91 (96)	98 (164)	96 (177)	95 (86)
		Travel-A	69 (9)	55 (23)	60 (31)	51 (20)	32 (25)	26 (5)	38 (6)	39 (15)	44 (19)	39 (11)
	*bla*_*CTX-M*_	GBMSM-A		50 (1)	0 (0)	0 (0)	3 (1)	1 (1)	0(0)	4 (6)	2 (4)	2 (2)
		Travel-A	0 (0)	2 (1)	8 (4)	0(0)	1 (1)	5 (1)	31 (5)	16 (6)	7 (3)	7 (2)
	*bla*_*TEM-1*_	GBMSM-A		0 (0)	0 (0)	67 (2)	18 (6)	4 (3)	8 (8)	9 (15)	14 (25)	45 (41)
		Travel-A	31 (4)	24 (10)	29 (15)	21 (8)	22 (17)	26 (5)	38 (6)	53 (20)	53 (23)	46 (13)
Fluoroquinolones	*QnrS*	GBMSM-A		0 (0)	0 (0)	0 (0)	3 (1)	0 (0)	2 (2)	0 (0)	1 (1)	3 (3)
		Travel-A	31 (4)	17 (7)	29 (15)	21 (8)	18 (14)	21 (4)	44 (7)	47 (18)	44 (19)	29 (8)
	*gyrA*	GBMSM-A		0 (0)	0 (0)	0 (0)	0 (0)	0 (0)	0 (0)	0 (0)	0 (0)	0 (0)
		Travel-A	15 (2)	19 (8)	13 (7)	10 (4)	3 (2)	5 (1)	6 (1)	16 (6)	33 (14)	25 (7)
	*parC80*	GBMSM-A		0 (0)	0 (0)	0 (0)	0 (0)	0 (0)	0 (0)	0 (0)	0 (0)	0 (0)
		Travel-A	0 (0)	2 (1)	0 (0)	0 (0)	1 (1)	0 (0)	0 (0)	13 (5)	19 (8)	21 (6)
Macrolides	*ermB*	GBMSM-A		0 (0)	0 (0)	67(2)	18 (6)	3 (2)	8 (8)	11(18)	19 (36)	41 (37)
		Travel-A	0 (0)	2 (1)	2 (1)	0 (0)	1 (1)	0 (0)	0 (0)	0 (0)	0 (0)	4 (1)
	*mphA*	GBMSM-A		0 (0)	0 (0)	67 (2)	21(7)	4 (3)	9 (9)	13 (22)	19 (36)	40 (36)
		Travel-A	0 (0)	2 (1)	8 (4)	8 (3)	8 (6)	5 (1)	6 (1)	8 (3)	19(8)	14(4)
Diaminopyrimidines	*dfrA1*	GBMSM-A		100 (2)	100 (3)	100 (3)	100 (33)	97 (74)	95 (100)	99 (166)	97 (180)	97 (88)
		Travel-A	62 (8)	40 (17)	46 (24)	36 (14)	23 (18)	11 (2)	63 (10)	42 (16)	51 (22)	36 (10)
Tetracyclines	*tet*	GBMSM-A		100 (2)	100 (3)	100 (3)	100 (33)	97 (74)	95 (100)	99 (166)	97 (180)	96 (87)
		Travel-A	85 (11)	79 (33)	87 (45)	74 (29)	78 (60)	95 (18)	88 (14)	74 (28)	70 (30)	68 (19)
Sulfonamides	*sul1*	GBMSM-A		0 (0)	0 (0)	0 (0)	3 (1)	1 (1)	2 (2)	4 (6)	1 (2)	3 (3)
		Travel-A	0 (0)	7 (3)	4 (2)	5 (2)	5 (4)	0 (0)	0 (0)	5 (2)	7 (3)	4 (1)
	*sul2*	GBMSM-A		100 (2)	33 (1)	33 (1)	58 (19)	66 (50)	55 (58)	53 (89)	31(57)	14(13)
		Travel-A	62 (8)	48 (20)	52 (27)	36 (14)	31 (24)	21(4)	31(5)	42 (16)	49 (21)	39 (11)
Amphenicols	*CatA1*	GBMSM-A		100 (2)	100 (3)	100 (3)	100 (33)	97 (74)	96 (101)	98 (165)	97 (180)	97 (88)
		Travel-A	77 (10)	55 (23)	62 (32)	51 (20)	38 (29)	21 (4)	25 (4)	26 (10)	40 (17)	39 (11)

### Multiple isolates from the same patient in GBMSM-A clades vs. Travel-A lineages

Repeat isolations were more frequently observed in the GBMSM-A clades [14.6% (*n*=97/666, isolated from 47 individuals)] than the Travel-A lineages [6% (*n*=22/372, isolated from 10 individuals)] (two-tailed *P*<0.001). WGS showed that all isolates from the same patient fell within the same 10-SNP single-linkage cluster, likely indicative of extended shedding rather than multiple infections. This was true for both GBMSM-A clades and Travel-A lineages.

## Discussion

The increase in notifications of *S. flexneri* 1b from 2018 to the first quarter of 2024 was attributed to domestically acquired infection in adult males. Previous studies have shown that this demographic picture is most commonly associated with sexual transmission among GBMSM [6,10, 26]. Travel-associated infections continued to occur; however, the travellers exhibited different demographic characteristics with respect to age and gender from those cases associated with sexual transmission, with a higher proportion of females and children.

We constructed a phylogeny of the *S. flexneri* 1b isolates annotated with epidemiological data and the presence of AMR determinants and identified a distinct clade of phylogenetically closely related isolates, with over 90% belonging to the same 10-SNP single-linkage cluster. This clade was characterized by a high proportion of adult males who had limited reports of travel to regions high-risk for *Shigella*. Outside this clade, isolates were more phylogenetically diverse, there was a higher proportion of women and children, and a higher proportion of cases reported recent travel to high-risk regions. Both the genomic and epidemiological analyses indicate that the recent increase in notifications of *S. flexneri* 1b in the UK was most likely caused by sexual transmission of a recently emerged strain among GBMSM.

Between 2009 and 2018, we observed four overlapping epidemics of shigellosis caused by *S. flexneri* 3a, *S. flexneri* 2a, *S. sonnei* clade 2 (genotype 3.7.29.1.2) and *S. sonnei* clade 5 (genotype 3.6.1.1.2) [7,27,29]. Just prior to the SARS-CoV-2 pandemic in March 2020, the dominant strains were *S. sonnei* clade 5 and a novel variant of *S. flexneri* 2a, although *S. flexneri* 1b and *S. flexneri* 3a were also detected [30]. During the pandemic, notifications of shigellosis rapidly declined [4], although diagnoses of *S. flexneri* 2a and 1b quickly returned to pre-pandemic levels. Post-pandemic, this trend continued. To date, the *S. flexneri* 1b epidemic strain has been circulating in the GBMSM community for over 6 years, since 2019. The pattern of shifting dominance of *Shigella* species, serotypes and clades in GBMSM in England, manifesting as successive waves, may indicate negative frequency-dependent selection mediated by host immunity and other, unknown, factors [16]. Since the SARS-CoV-2 pandemic, this pattern is less obvious: different serotypes appear to be circulating concurrently [30].

For the most part, the emergence of each epidemic has corresponded with the acquisition of AMR determinants, with the early epidemic strains all sequentially acquiring pKSR100-like plasmids carrying genes conferring resistance to azithromycin. Baker *et al.* showed that pKSR100-like plasmids were transmitted between *S. flexneri*, *S. sonnei* and an atypical Shiga toxin-producing *Escherichia coli* serotype, O117:H7 [17]. Epidemics of sexually transmitted shigellosis are characteristically associated with resistance to macrolides, fluoroquinolones and, more recently, third-generation cephalosporins. The emergence and persistence of ESBL-producing *S. sonnei*, harbouring *bla*_CTX-M_ variants, *bla*_CTX-M27_ and subsequently *bla*_CTX-M15_, have caused a European-wide outbreak. These XDR strains have continued to circulate across Europe, suggesting clonal persistence beyond the initial outbreak period [31,34].

In contrast to the MDR and XDR epidemic strains of *S. flexneri* and *S. sonnei* described above, *S. flexneri* 1b characteristically carries AMR genes conferring resistance to trimethoprim, tetracycline, chloramphenicol and the second-generation beta-lactams; the latter three of which are typically conferred by the *Shigella* resistance locus and have previously been associated as precursors to the emergence of GBMSM-associated epidemics of *S. flexneri* 3a and *S. flexneri* 2a. Despite the co-circulation of *S. flexneri* 1b among GBMSM with epidemic strains of *S. flexneri* and *S. sonnei*, less than 10% of GBMSM-A *S. flexneri* 1b have acquired resistance to the macrolides, fluoroquinolones or third-generation cephalosporins, suggesting other factors are at play.

Unlike previous epidemics of sexually transmitted shigellosis, the emergence of the *S. flexneri* 1b GBMSM-A clade was not characterized by rapid, widespread acquisition of the pKSR100-like plasmid or other azithromycin resistance determinants. From the ecological perspective, it is possible that azithromycin selection pressure has recently abated for sexually transmissible *Shigella* in the UK. Previously, the use of macrolides for the first-line treatment of sexually transmitted bacterial infection, gonorrhoea, drove the emergence of azithromycin resistance in *Neisseria gonorrhoea* and *Shigella* species, the latter as bystander resistance. Recently, treatment regimens for gonorrhoea were updated and switched from macrolides to ceftriaxone, and ongoing bystander resistance is evidenced in *Shigella* species by the emergence of ESBL-producing strains. From the pathogen perspective, among the strains of *S. flexneri* 1b in the GBMSM-A clade, the fitness advantage associated with acquisition of the pKSR100-like plasmid harbouring resistance to *mphA*, *ermB* and *bla_TEM-1_* may be negated by the presence of *bla_OXA-1_* chromosomal gene. The *bla_OXA-1_* gene, which is highly conserved in *S. flexneri* 1b, confers resistance to a similar substrate profile to that of *bla_TEM-1_* and may reduce the need for acquisition of the pKSR100-like plasmid. It has been suggested that a lack of immunity to newly emerging types might be a contributing factor facilitating transmission; however, currently, there is little evidence in the literature to support this speculation. Prior to 2019, *S. flexneri* 1b was relatively uncommon generally and rarely found among the GBMSM community, and, therefore, the population was susceptible to infection with this type.

Antimicrobial tolerance and persistence are thought to be precursor phenotypes for the acquisition of AMR. Recent studies revealed an association of MDR in *S. sonnei* with multi- and highly variable copies of *metG*, borne on a phage-plasmid [35]. The expression of the phage-plasmid encoded *metG* created a sub-population of bacteria that predisposed them to the acquisition of resistance to third-generation cephalosporins. Phage-plasmid encoded *metG* was not detected in the *S. flexneri* 1b isolates in this study.

A notable limitation of this study was the lack of information about sexual orientation and the incomplete travel histories. Adult male case-patients who may have travelled were categorized as presumptive MSM within this cluster if the travel histories were not recorded. Identifying as GBMSM and reporting recent travel are also not mutually exclusive; therefore, there are limitations with the use of the presumptive MSM proxy definition.

Sexual transmission among GBMSM continues to drive notifications of *S. flexneri* in England. Historically, we have observed specific GBMSM epidemic clades of *S. flexneri* and *S. sonnei* rise and fall over a 3- to 4-year time frame, whereas, currently, the GBMSM *S. flexneri* 1b clade has been circulating for 6 years. Despite this persistent phenotype, the GBMSM *S. flexneri* 1b clade is not associated with the same characteristics that drive the transmission of isolates belonging to previously described GBMSM clades, specifically plasmid-encoded resistance to azithromycin and third-generation cephalosporins, the acquisition of mutations encoding resistance to fluoroquinolones and phage-plasmid-encoded *metG*. Clearly, drivers for the emergence and persistence of infection are multifactorial, involving characteristics of the environment, the pathogen and/or the host. Intervention strategies should take into consideration that the emergence and persistence of *Shigella* species, serotypes and clades circulating concurrently in the GBMSM community may be influenced by a variety of different factors, from resistance to current antimicrobial treatment regimens to immunologically naïve hosts.

## Supplementary material

10.1099/mgen.0.001495 Uncited Fig. S1.

## Abbreviations

ARG: antimicrobial resistance genes
CI: confidence interval
COVID: coronavirus disease
GBMSM: gay, bisexual and other men who have sex with men
GBMSM-A: GBMSM-associated
GBRU: Gastrointestinal Bacteria Reference Unit
GDW: Gastrointestinal Data Warehouse
IQR: interquartile range
MDR: multidrug resistant
MSM: men who have sex with men
OR: odds ratio
SARS-CoV-2: severe acute respiratory syndrome coronavirus 2
Travel-A: travel-associated
UKHSA: UK Health Security Agency
WGS: whole-genome sequencing
XDR: extensively drug-resistant
